# Subarachnoid Hemorrhage Induces Dynamic Immune Cell Reactions in the Choroid Plexus

**DOI:** 10.3389/fncel.2020.00018

**Published:** 2020-02-11

**Authors:** Peter Solár, Ilona Klusáková, Radim Jančálek, Petr Dubový, Marek Joukal

**Affiliations:** ^1^Department of Anatomy, Cellular and Molecular Neurobiology Research Group, Faculty of Medicine, Masaryk University, Brno, Czechia; ^2^Department of Neurosurgery, Faculty of Medicine, Masaryk University and St. Anne’s University Hospital Brno, Brno, Czechia

**Keywords:** subarachnoid hemorrhage, choroid plexus, intracranial hypertension, macrophages, blood-cerebrospinal fluid barrier

## Abstract

Subarachnoid hemorrhage (SAH) is a specific form of hemorrhagic stroke that frequently causes intracranial hypertension. The choroid plexus (CP) of the brain ventricles is responsible for producing cerebrospinal fluid and forms the blood – cerebrospinal fluid barrier. The aim of the current study was to determine whether SAH induces an immune cell reaction in the CP and whether the resulting increase in intracranial pressure (ICP) itself can lead to cellular changes in the CP. SAH was induced by injecting non-heparinized autologous blood to the cisterna magna. Artificial cerebrospinal fluid (ACSF) instead of blood was used to assess influence of increased ICP alone. SAH and ACSF animals were left to survive for 1, 3, and 7 days. SAH induced significantly increased numbers of M1 (ED1+, CCR7+) and M2 (ED2+, CD206+) macrophages as well as MHC-II+ antigen presenting cells (APC) compared to naïve and ACSF animals. Increased numbers of ED1+ macrophages and APC were found in the CP only 3 and 7 days after ACSF injection, while ED2+ macrophage number did not increase. CD3+ T cells were not found in any of the animals. Following SAH, proliferation activity in the CP gradually increased over time while ACSF application induced higher cellular proliferation only 1 and 3 days after injection. Our results show that SAH induces an immune reaction in the CP resulting in an increase in the number of several macrophage types in the epiplexus position. Moreover, we also found that increased ICP due to ACSF application induced both an immune reaction and increased proliferation of epiplexus cells in the CP. These findings indicate that increased ICP, and not just blood, contributes to cellular changes in the CP following SAH.

## Introduction

Subarachnoid hemorrhage (SAH) is a specific form of hemorrhagic stroke that is recorded in up to 10 cases per 100,000 people annually, and has a combined morbidity and mortality of more than 50% (King, 1997; Rincon et al., 2013). More than 80% of these cases are due to the rupture of a cerebral aneurysm with bleeding into the subarachnoid cisterns and fissures (Fisher et al., 1980; van Gijn and Rinkel, 2001). In the first few seconds following aneurysm rupture, intracranial pressure (ICP) rises rapidly and brain tissue gets compressed. This sudden rise in ICP may reach values up to diastolic blood pressure but later falls to slightly above the baseline (Prunell et al., 2003).

The extent to which ICP rises is correlated with early onset brain injury and mortality (Zoerle et al., 2015). The inflammatory cascade that gets activated within minutes after SAH comprises adhesion molecules, cytokines, migration of leukocytes, and complement activation (Sehba et al., 2011). Early inflammation seems to play an important role in development of delayed cerebral ischemia and affects the outcome after SAH (Sercombe et al., 2002; Miller et al., 2014).

The choroid plexus (CP) of the brain ventricles is responsible for producing almost 60 – 80% cerebrospinal fluid (CSF) by volume. The CP comprises a highly vascularized stroma containing loose connective tissue, fenestrated capillaries and immune cells. The stroma is covered by a layer of cuboidal epithelial cells linked by tight junctions that is one of the fundamental components of the blood–cerebrospinal fluid (B-CSF) barrier. Kolmer cells (KC) of the epiplexus adhere to the ventricular side of these cuboidal epithelial cells and are in direct contact with CSF (Hosoya and Fujita, 1973; Segal and Pollay, 1977; McMenamin et al., 2003; Wolburg and Paulus, 2009).

There is a growing body of evidence that implicates changes in the CP (due to extravasated blood into the CSF following SAH) in hypersecretion of CSF leading to the development of post-hemorrhagic hydrocephalus (Simard et al., 2011; Xiang et al., 2017). It is believed that post-hemorrhagic hydrocephalus results when CSF flow within the ventricular system is impaired or there is a structural blockage of CSF drainage in the arachnoid villi (Yong et al., 2010). It has been suggested that SAH may also lead to hypersecretion of CSF by the CP. Aydin et al. (2014) showed that increased CSF secretion seems to be triggered by the petrous ganglion of the glossopharyngeal nerve and the numerous water-filled vesicles in the early stages of SAH. Similarly, Kanat et al. (2013) observed increased CSF secretion caused by fluid filled vesicles in the CP following SAH and concluded that hypersecretion of CSF contributes to the development of post-hemorrhagic hydrocephalus.

It has been found that SAH increases the number of macrophages in the epiplexus position of the CP (Liszczak et al., 1984). However, the type of macrophages and other immune cells that increase and proliferate after SAH remains a mystery. Further, the dynamics of these cell changes is also unclear. There is very little known about the effect of elevated ICP and inflammation on the SAH-affected CP.

The aim of our study was to investigate and specify cellular changes in the CP following SAH by monitoring the different subpopulations of macrophages, antigen presenting cells (APC), T lymphocytes as well as cellular proliferation over time. A further objective was to clarify whether these changes are caused mainly by blood degradation products or whether increased intracranial pressure after SAH also has an effect. We found that SAH induced increases in the number of several subpopulations of immune cells predominantly in the epiplexus position and enhanced their proliferation in the CP. Moreover, artificial cerebrospinal fluid (ACSF) application also induced similar increases in immune cell numbers in the CP. The results indicate that immune cell changes in the CP can be induced not only by blood and blood degradation products but also by increased ICP.

## Materials and Methods

### Animals and Surgical Procedures

Experiments were performed on 54 adult male rats (Wistar 200 – 250 g; Animal Breeding Facility, Masaryk University, Czechia). All experimental procedures were carried out aseptically and according to protocols approved by the Ethical Committee of Masaryk University, Brno and the Departmental Committee of the Ministry of Education, Youth and Sports, Czechia. The rats were anesthetized with a mixture of 5% ketamine (100 mg/kg) and 2% xylazine (10 mg/kg) administrated intraperitoneally.

Subarachnoid hemorrhage was induced by the application of non-heparinized autologous arterial blood into the cisterna magna following the standardized method originally published by Solomon et al. (1985) and modified by other authors (d’Avella et al., 1996; Matz et al., 1996). Briefly, cannulation of the caudal artery was performed (SAH group) and a midline suboccipital incision was made to expose the arch of the atlas, the occipital bone and the atlanto-occipital membrane. Then, the animal was placed in a stereotaxic apparatus (Kopf Instruments, Tujunga, CA, United States), the atlanto-occipital membrane was cleared of connective tissue, and a syringe with 30G needle was placed on the manipulating arm of the stereotaxic frame tilted at 60° from horizontal plane. Under the high magnification of a surgical microscope the arachnoid was penetrated by the needle and 200 μl of non-heparinized autologous blood taken from the caudal artery was injected within 60 s into the cisterna magna (SAH group).

Two hundred μl of ACSF containing 130 mmol/l NaCl, 3.0 mmol/l KCl, 1.2 mmol/l NaH_2_PO_4_, 20 mmol/l NaHCO_3_, 1.3 mmol/l MgCl_2_, 2.4 mmol/l CaCl_2_, 10 mmol/l Glucose (Hylden and Wilcox, 1980) instead of blood was injected within 60 s into the cisterna magna to the animals of ACSF group.

The needle was slowly withdrawn 2 min after injection to prevent leakage of blood or ACSF from the cisterna magna and the membrane puncture was closed by gelatin sponge. The muscles and the skin were closed by 4-0 silk suture.

Rats exposed to blood (SAH group) or ACSF (ACSF group) were left to survive for 1, 3, and 7 days (*n* = 8 SAH; *n* = 8 ACSF for each time point).

Rats of SAH and ACSF groups as well as a naive group (*n* = 6) were sacrificed using CO_2_, perfused transcardially with 500 ml heparinized (1000 units/500 ml) phosphate–buffered saline (PBS; pH 7.4) followed by 500 ml of Zamboni’s fixative (Zamboni and de Martino, 1967).

Then, brains were promptly removed and assessed for successful injection. In the SAH group, brains from animals with expressed SAH (presence of the blood in the subarachnoid cisterns and basal surface of the brain after full blood volume injection) and brains with no SAH from the ACSF group (no blood in the subarachnoid space, full ACSH volume injection) were included. After macroscopic assessment, the brains were immersed in Zamboni’s fixative for 3 days, washed in 10% sucrose and embedded in Tissue-Tek OCT compound (Miles, Elkhart, IN, United States). Coronal cryostat sections were cut (20 μm sections; Leica 1800 cryostat; Leica Microsystem, Wetzlar, Germany) and mounted onto chrome-alum covered microscope slides.

### Immunohistochemical Staining

To detect subpopulations of macrophages, the brain sections of naive, SAH and ACSF groups were immunostained under identical conditions with anti-CD68 (activated ED1+ macrophages), anti-CD163 (resident ED2+ macrophages), anti-CCR7 and anti-CD206 antibodies. M1 macrophages are characterized by expression of CD68 (ED1) and CCR7 besides other antigens (Badylak et al., 2008; Ma et al., 2010). The expression of CD163 (ED2) and CD206 antigens marks the M2 macrophage phenotype (Kigerl et al., 2009). APC were detected using an anti-major histocompatibility complex class II (MHC II) antibody (Dijkstra and Damoiseaux, 1993), and T lymphocytes with anti-CD3 antibody (Mason et al., 1989). Proliferation activity was assessed using Ki-67 immunostaining (Gerdes et al., 1984).

Sections were washed with PBS containing 0.3% bovine serum albumin and 0.1% Tween-20, treated with 3% normal serum for 30 min. and then incubated with the primary antibody at room temperature. Primary antibody sources and incubation conditions are listed in Table 1. Affinity purified Cy5- or FITC conjugated donkey anti-rabbit or anti-mouse secondary antibodies (Jackson, 1:100) were applied at room temperature for 90 min. Control sections were incubated in parallel omitting the primary antibodies. Positive immunostaining of CD3 antibody was confirmed in sections of the spleen. Immunostained sections were rinsed, stained with Hoechst 33342 (Sigma, St. Louis, MO, United States) to locate cell nuclei, and mounted in Vectashield aqueous mounting medium (Vector Laboratories, Burlingame, CA, United States). Immunofluorescence was observed and analyzed using a Nikon Eclipse NI-E epifluorescence microscope, equipped with a Nikon DS-Ri1 camera (Nikon, Prague, Czechia).

**TABLE 1:** List of primary antibodies used, their dilutions, incubation times and suppliers.

Name	Type of antibody	Dilution	Incubation time	Supplier
ED1	Mouse monoclonal	1:200	240 min	Serotec
ED2	Mouse monoclonal	1:200	16 hrs	Serotec
Ki-67	Rabbit polyclonal	1:500	240 min	Vector Laboratories
MHC II	Mouse monoclonal	1:100	Overnight	Serotec
CCR7	Rabbit polyclonal	1:50	Overnight	Acris
CD206	Rabbit polyclonal	1:50	Overnight	Santa Cruz
CD3	Rabbit monoclonal	1:100	Overnight	Abcam

### Double Immunohistochemical Staining

Proliferation activity of APC and macrophages in the CP was monitored using double immunostaining for MHC II, ED1 or ED2 and Ki-67. A subset of sections was incubated with rabbit polyclonal anti-Ki-67 antibody and treated with FITC-conjugated donkey anti-rabbit secondary antibody for 90 min. After thorough washing, primary MHC II, ED1 or ED2 antibody was applied overnight followed by Cy5-conjugated secondary antibody. Sections were mounted and analyzed as above.

### Image and Statistical Analysis

Images (magnification 200x) were acquired from at least 10 sections cut at 60 μm intervals through the brain ventricles containing the CP for each group of animals. CP area was determined in the images, manually edited when needed, and measured using the NIS-Elements AR Analysis software (version 4.20.00, Nikon, Prague, Czechia). ED1, ED2, CCR7, CD206, MHC II, and CD3 immunopositive cells (ED1+, ED2+, CCR7+, CD206+, MHC II+, and CD3+) were correlated using the positions of cell nuclei and counted manually in the defined CP area. The analysis was performed in a randomized fashion by an investigator who was blinded to the group of animals. The number of positive cells for every 1 mm^2^ of CP area was expressed as mean ± standard deviation (SD).

To detect cellular proliferation, double immunostaining (ED1, ED2 or MHC II and Ki67) was analyzed in the colocalization module of NIS Elements software (Nikon, Czechia). The percentage of ED1+/Ki-67+, ED2+/Ki-67+, and MHC II+/Ki-67+ cells (to total Ki-67+ cell number) was expressed as mean ± standard deviation (SD).

The data of naive, ACSF and SAH groups were compared pairwise using the Mann–Whitney *U*-test in STATISTICA 5.5 software (StatSoft, Tulsa, OK, United States).

## Results

All animals that underwent application of blood (SAH group; *n* = 24) or ACSF (*n* = 24) survived the experiments and the injection was successful (scored as the presence of blood in the subarachnoid space in the SAH group and no blood in the ACSF group).

### Position and Number of Immune Cells in the CP

**FIGURE 1:**
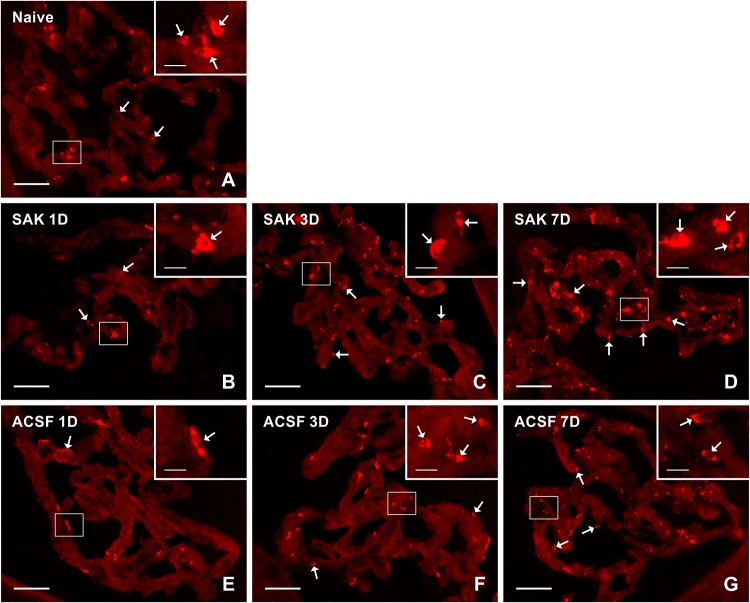
Representative pictures showing results of immunostaining with ED1 antibody in cryostat sections of the CP from naïve **(A)**, SAH **(B–D),** and ACSF **(E–G)** rats at 1, 3 and 7 days (1D, 3D, and 7D) after operation. SAH induced an increase in the number of ED1+ cells 3 and 7 days after operation. Increased numbers of ED1+ cells were also found 3 and 7 days after application of ACSF. Arrows indicate ED1+ cells predominantly in the epiplexus position of the CP. Insets show a higher magnification of regions marked by the boxes. Scale bars = 80 μm (main image); 10 μm (insets).

**FIGURE 2:**
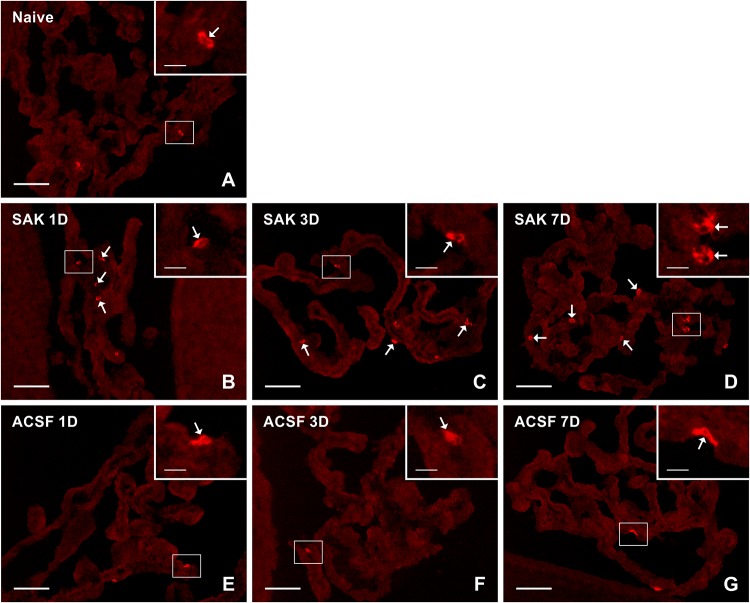
Representative pictures of cryostat sections through the CP showing immunostaining with ED2 antibody from naïve **(A)**, SAH (**B–D**), and ACSF **(E–G)** rats at 1, 3 and 7 days (1D, 3D, and 7D) after operation. SAH led to increased numbers of ED2+ cells at 1, 3, and 7 days after operation. The number of ED2+ cells in CP of ACSF animals did not increase at any time point after ACSF administration. Arrows indicate ED2+ cells in the epiplexus position of the CP. Insets show a higher magnification of regions marked by the boxes. Scale bars = 80 μm (main image); 10 μm (insets).

**FIGURE 3:**
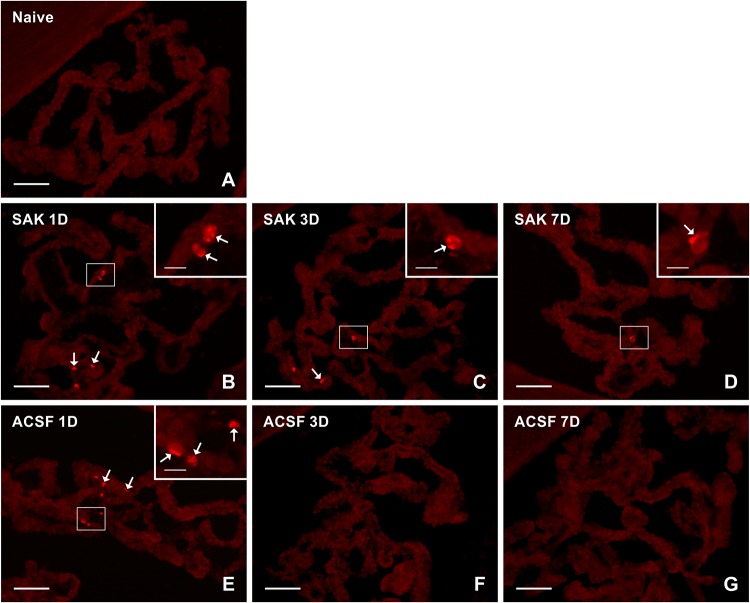
Representative pictures showing results of immunostaining with CCR7 antibody in cryostat sections of the CP from naïve **(A)**, SAH **(B–D)**, and ACSF **(E–G)** rats at 1, 3 and 7 days (1D, 3D, and 7D) after operation. The number of CCR7+ cells was increased 1 and 3 days following induction of SAH and 1 day after ACSF administration. Arrows indicate CCR7+ cells in the epiplexus position of the CP. Insets show a higher magnification of regions marked by the boxes. Scale bars = 80 μm (main image); 10 μm (insets).

**FIGURE 4:**
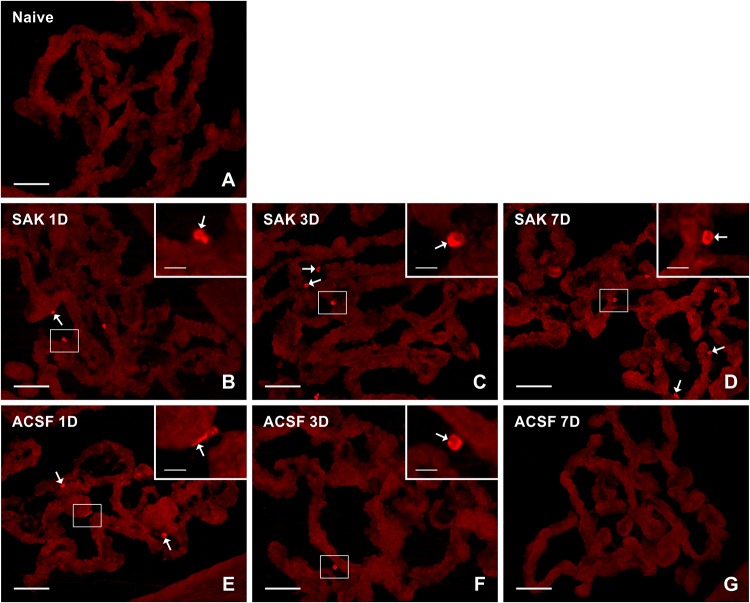
Representative pictures of cryostat sections through the CP showing immunostaining with CD206 antibody from naïve **(A)**, SAH **(B–D),** and ACSF **(E–G)** rats at 1, 3, and 7 days (1D, 3D, and 7D) after operation. Increased numbers of CD206+ cells were found 1, 3, and 7 days following induction of SAH. ACSF application led to an increase in the number of CD206+ cells 1 and 3 days following injection. Arrows indicate CD206+ cells in epiplexus position of the CP. Insets show a higher magnification of regions marked by the boxes. Scale bars = 80 μm (main image); 10 μm (insets).

Activated (ED1+) and resident (ED2+) macrophages as well as CCR7+ and CD206+ cells were found mainly on the ventricular side of the cuboidal epithelial cells in the epiplexus position (Figures 1–4).

**FIGURE 5:**
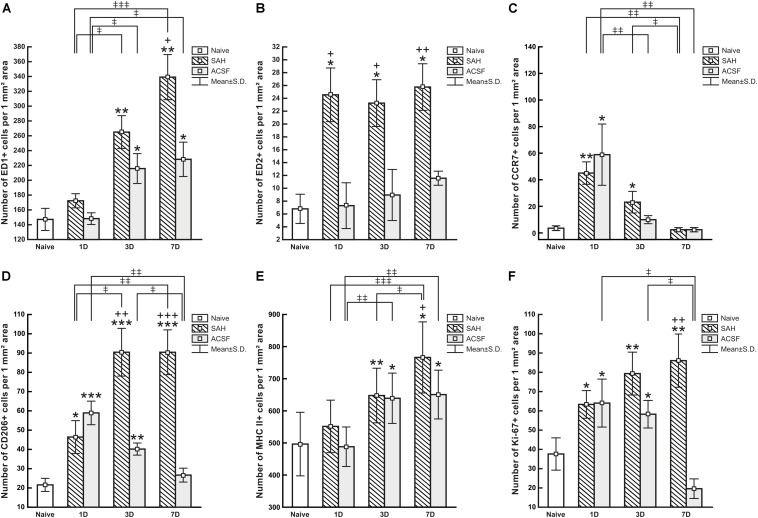
Numbers of ED1+ **(A)**, ED2+ **(B)**, CCR7+ **(C)**, CD206+ **(D)**, MHC II+ **(E)** and Ki67+ **(F)** cells per mm^2^ of CP from naive, ACSF and SAH rats at 1, 3, and 7 days after operation. Error bars indicate SD. *Indicates a significant difference when compared to CP from naïve rats (*p < 0.05; **p < 0.01; ***p < 0.001). +Indicates a significant difference when compared to CP from ACSF rats (^+^*p* < 0.05; ^++^*p* < 0.01; ^+++^*p* < 0.001). ‡Indicates a significant difference between the individual time points of survival (^‡^*p* < 0.05; ^‡‡^*p* < 0.01; ^‡⁣‡‡^*p* < 0.001).

The number of ED1+ macrophages in the CP of SAH and ACSF animals increased over time in our experiments. One day after SAH, the numbers of ED1+ macrophages in the CP was slightly increased compared to CP of naïve control (*n* = 147.2 ± 36.5/mm^2^). A significant increase, compared to the CP of naïve rats, in the number of ED1+ macrophages was found 3 days (*n* = 265 ± 70/mm^2^; *p* < 0.01) and 7 days (*n* = 339.1 ± 85.9/mm^2^; *p* < 0.01) after SAH as well as 3 days (*n* = 215.8 ± 49.4/mm^2^; *p* < 0.05) and 7 days (*n* = 228.2 ± 51.8/mm^2^; *p* < 0.05) after ACSF injection. The number of ED1 + macrophages after 7 days was significantly higher in the CP of the SAH animals (*n* = 339.1 ± 85.9/mm^2^; *p* < 0.05) compared to the ACSF group (*n* = 228.2 ± 51.8/mm^2^; Figure 5A).

Immunoquantification showed a similar significant increase in the number of ED2+ macrophages at 1 (*n* = 24.5 ± 9.3/mm^2^; *p* < 0.05), 3 (*n* = 23.3 ± 1.5/mm^2^; *p* < 0.05) and 7 days (*n* = 25.8 ± 9.6/mm^2^; *p* < 0.05) following SAH compared to naïve rats (*n* = 6.8 ± 4.5/mm^2^). Strikingly, the number of ED2+ macrophages was significantly increased 1, 3, and 7 days following SAH, also when compared to ACSF animals at the same time points (*n* = 7.3 ± 7.9/mm^2^; *n* = 8.85 ± 4.95/mm^2^; *n* = 11.5 ± 2.4/mm^2^, respectively). No significant changes in the number of ED2+ cells were found in the CP of the ACSF animals when compared to naïve controls (Figure 5B).

The number of CCR7+ cells was significantly increased, compared to CP of naïve controls, in the CP of animals 1 (*n* = 45 ± 8.4/mm^2^; *p* < 0.01) and 3 days (*n* = 23.1 ± 8.1/mm^2^; *p* < 0.05) following SAH (*n* = 3.6 ± 1.8/mm^2^). The number of CCR7+ cells in the CP of the SAH and ACSF group decreased over time and dropped to normal at 7 days. Moreover, the CP of the ACSF group showed a significantly higher CCR7 + cell number (*n* = 58.9 ± 23/mm^2^; *p* < 0.05), compared to CP of naïve rats only after 1 day following ACSF application (*n* = 3.6 ± 1.8/mm^2^; Figure 5C).

Immunostaining showed a significantly higher number, compared to naïve controls (n = 21.6 ± 3.4/mm^2^), of anti-inflammatory CD206+ cells in the CP 1 (*n* = 46.4 ± 8.5/mm^2^; *p* < 0.05), 3 (*n* = 90.5 ± 12.4/mm^2^; *p* < 0.001) and 7 days (*n* = 90.4 ± 11.6/mm^2^; *p* < 0.001) after SAH. However, the number of CD206 + cells in the CP of the ACSF group decreased over time following ACSF injection; it was significantly increased compared to CP of naïve animals (*n* = 21.6 ± 3.4/mm^2^) at 1 (*n* = 58.9 ± 6.1/mm^2^; *p* < 0.001) and 3 days (*n* = 40.2 ± 3.2/mm^2^; *p* < 0.01), but dropped to normal at 7 days. SAH significantly increased the number of CD206+ cells in the CP 3 (*n* = 90.5 ± 12.4/mm^2^; *p* < 0.01) and 7 days (*n* = 90.4 ± 11.6/mm^2^; *p* < 0.001) when compared to ACSF at the same time points (*n* = 40.2 ± 3.1/mm^2^ and *n* = 26.7 ± 3.6/mm^2^, respectively; Figure 5D).

**FIGURE 6:**
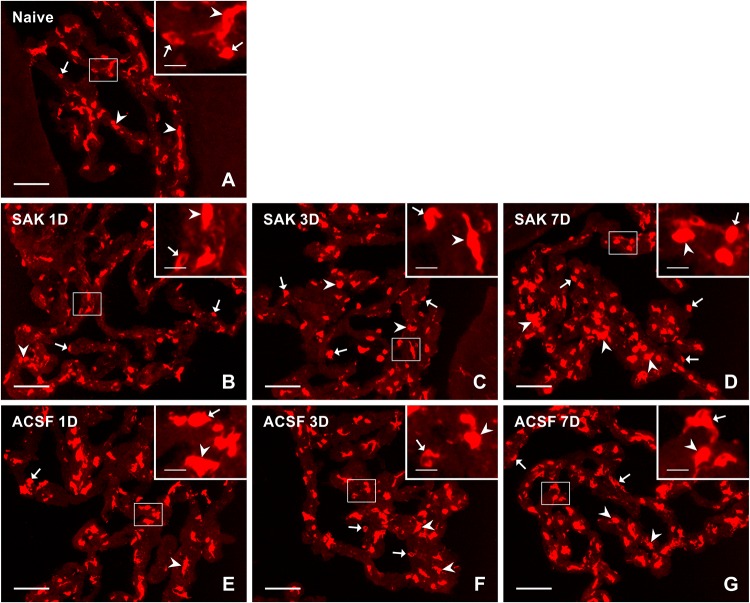
Representative pictures of cryostat sections through the CP showing immunostaining with MHC II antibody from naïve **(A)**, SAH **(B–D),** and ACSF **(E–G)** rats at 1, 3, and 7 days (1D, 3D, and 7D) after operation. Arrows indicate MHC II+ cells in the epiplexus position and arrowheads in the stroma of CP. Insets show a higher magnification of regions marked by the boxes. Scale bars = 80 μm (main image); 10 μm (insets).

MHC II+ APC displayed a range of shapes from oval to ramified and were found in the epiplexus position as well as in the stromal core of the CP (Figure 6). The APC population of the CP showed a progressive increase in number following SAH over the duration of our experiment. Significantly increased numbers of APC were found 3 (*n* = 647.6 ± 85.1/mm^2^; *p* < 0.01) and 7 days (*n* = 766.2 ± 111.1/mm^2^; *p* < 0.05) following SAH as well as 3 (*n* = 639.5 ± 77.6/mm^2^; *p* < 0.05) and 7 days (*n* = 651 ± 75.6/mm^2^; *p* < 0.05) following ACSF injection when compared to naïve rats (*n* = 495.7 ± 98.7/mm^2^). The number of MHC II+ APC in the CP was significantly increased 7 days (*n* = 766 ± 111.1/mm^2^; *p* < 0.05) after SAH compared to ACSF animals (*n* = 651 ± 75.6/mm^2^; Figure 5E). CD3 immunostaining did not reveal T cells in the CP of any group of the animals (not shown).

### Assessment of Proliferation Activity in the CP

**FIGURE 7:**
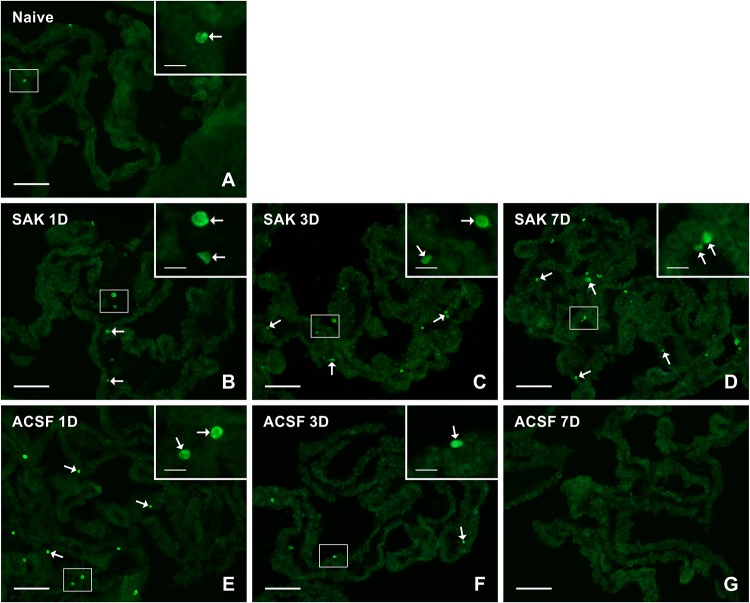
Representative pictures of cryostat sections through the CP showing immunostaining with Ki67 antibody from naïve **(A)**, SAH **(B–D)**, and ACSF **(E–G)** rats at 1, 3, and 7 days (1D, 3D, and 7D) after operation. SAH induced a progressive increase in the number of proliferating cells 1, 3, and 7 days after injection of blood. ACSF application led to an increase in the number of KI67+ cells 1 and 3 days after operation. Arrows indicate Ki67+ cells in the epiplexus position of the CP. Insets show a higher magnification of regions marked by the boxes. Scale bars = 80 μm (main image); 10 μm (insets).

Immunostaining for Ki-67 revealed proliferating cells in the epiplexus position (Figure 7). Significantly increased proliferation of epiplexus KC was found 1 (*n* = 63.3 ± 6.8/mm^2^; *p* < 0.05), 3 (*n* = 79.3 ± 12.3/mm^2^; *p* < 0.01) and 7 days (*n* = 86.1 ± 13.8/mm^2^; *p* < 0.05) following SAH, compared to the number of Ki-67 positive cells in the CP of naïve animals (*n* = 37.6 ± 9.4/mm^2^). Moreover, the proliferation showed a tendency to increase. Significantly increased proliferation of KC was also found in the CP of the ACSF group 1 (*n* = 64 ± 12.6/mm^2^; *p* < 0.05) and 3 days (*n* = 58.2 ± 8.3/mm^2^; *p* < 0.05) after ACSF injection compared to naïve animals (*n* = 37.6 ± 9.4/mm^2^). KC proliferation activity returned to normal 7 days after ACSF application. The number of Ki-67+ cells was significantly increased 7 days (*n* = 86.1 ± 13.8/mm^2^; *p* < 0.01) after SAH compared to the control group 7 days after ACSF application (*n* = 19.6 ± 5.2/mm^2^) (Figure 5F).

**FIGURE 8:**
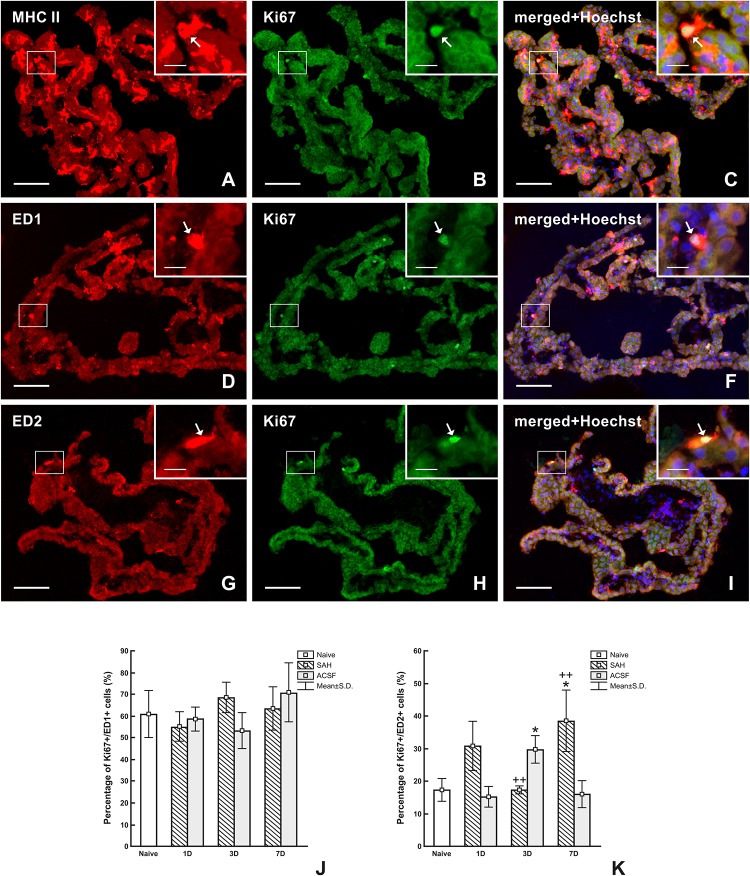
Representative images of double immunostaining to detect MHC II+, ED1+, and ED2+ macrophages as well as Ki67+ cells in the CP 7 days after SAH. Double immunostaining for MHC II and Ki-67 **(A–C)** showed that all proliferating cells are antigen presenting cells *(arrows)*. Simultaneous reaction for ED1 and Ki67 **(D–F)** proved that most proliferating cells are activated macrophages *(arrows)*. Double immunostaining reaction for ED2 and Ki-67 **(G–I)** showed that approximately one-third of proliferating cells are resident macrophages *(arrows)*. Merged figures **(C,F,I)** include a merge with Hoechst-stained nuclei. Scale bars – main images 80 μm; insets 20 μm. Percentage of ED1+/Ki-67+ cells **(J)** and ED2+/Ki67+ cells **(K)** in the CP of naïve, SAH and ACSF rats at 1, 3, and 7 days after operation. *Indicates a significant difference when compared to CP from naïve rats (*p* < 0.05). ^++^Indicates a significant difference when compared to CP from control rats (*p* < 0.01).

Double immunostaining of MHC II+ and Ki67+ cells revealed that all proliferating cells in the CP were APC in all animals (Figures 8A–C). Double immunostaining for ED1+ and Ki-67+ (Figures 8D–F) cells did not reveal significant changes in the percentage of proliferating activated macrophages in the CP following either SAH or ACSF application (Figure 8J). In contrast, double immunostaining for ED2+ and Ki-67+ cells (Figures 8G–I) displayed a significantly higher percentage of proliferating ED2+ cells 7 days (*n* = 38.4 ± 9.4%; *p* < 0.05) following SAH, but at 3 days (*n* = 17.2 ± 3.5%) the number was lower compared to ACSF animals. Surprisingly, the number of proliferating ED2+ cells in the CP was significantly higher, compared to naïve animals (*n* = 17.2 ± 3.5%), 3 days (*n* = 29.6 ± 4.2%; *p* < 0.05) after ACSF application. The proliferation of ED2+ cells was significantly increased 3 days following ACSF application (*n* = 29.6 ± 4.2/mm^2^; *p* < 0.01), when compared to 3 days after SAH induction (*n* = 17.2 ± 1.2%), whereas it was reversed at 7 days following SAH, being significantly higher (*n* = 38.4 ± 9.4%; *p* < 0.01) when compared to CP of the ACSF group (*n* = 15.9 ± 4.1%; Figure 8K).

## Discussion

### Subarachnoid Hemorrhage Induced Inflammatory Reaction in the Choroid Plexus

Subarachnoid hemorrhage from a ruptured intracranial aneurysm results in an initial increase of ICP which together with subsequent blood degradation products produce further clinical complications (Grote and Hassler, 1988; Kamp et al., 2013). The main pathophysiological changes like early brain injury, cerebrovascular inflammation and alteration/disturbance of the blood-brain barrier occur during the first several days after SAH (Germanò et al., 2000; Sercombe et al., 2002; Cahill et al., 2006).

In our experiments, we used the technique of volume infusion, a well-established model of SAH originally published by Solomon et al. (1985) where a constant volume of blood or ACSF is injected into the cisterna magna. Volume infusion is a standardized model with very low mortality in animals (0% in our experiments) and produces a similar change in ICP as well as in cerebral blood flow in all animals. It has been found that application of 200 μl of autologous arterial blood or ACSF into the subarachnoid cistern induced a sudden increase in ICP from 7 ± 1 to 107 ± 5 mmHg (102 ± 7 mmHg, respectively) within 15 s with a drop to near-normal levels within 2.5 min. Moreover, compared to other models, the volume infusion model is more valuable for late SAH changes (Prunell et al., 2002). We injected blood or ACSF within 60 s. It has been found that injection speed plays a crucial role in ICP dynamics, cerebral blood flow and autoregulation. Rapid injection (within 60 s), compared to slow injection (10, 30, or 60 min), produces the highest ICP peaks, autoregulation impairment and neuronal loss resulting in early brain injury (Conzen et al., 2019). In our experiments we did not assess behavioral changes because 7 days is too short a time for evaluating functional outcomes. Nevertheless, it has been found that blood injection into the cisterna magna induces motor impairment compared to saline injection (Germanò et al., 1994).

It is generally accepted that the products of heme catabolism, e.g., free ferrous iron, CO or biliverdin/bilirubin are biologically active substances that may exert profound effects on cellular differentiation, proliferation, physiology and death (Schipper, 2004). Therefore, it is reasonable to assume that the formation and subsequent degradation of a blood clot may lead to the alteration of macrophage numbers and increased cellular proliferation in the CP.

Our experimental study demonstrated that SAH induced an increase in the number of epiplexus macrophages with ED1, ED2, CCR7, CD206 and MHC immunophenotypes and caused their proliferation in the CP. These findings corroborate the findings of Liszczak et al. (1984), where increased KC numbers were seen in the CP 1 week after SAH, but they did not evaluate cellular specification, nor did they monitor earlier time points.

Immunostaining revealed higher numbers of activated (ED1+) macrophages and MHC II+ immune cells in the epiplexus position over the period of survival following SAH. This is probably linked to similar cellular specificity of the ED1 antibody and that recognizing MHC II. The ED1 antibody is produced against a 110-kD the lysosomal/endosomal-associated membrane glycoprotein (CD68) highly expressed by monocytes and tissue macrophages. This protein is a member of the scavenger receptor family involved in the clearance of cellular debris and the promotion of phagocytosis in activated macrophages (Juniantito et al., 2010; Zeibig et al., 2019). The ED1 monoclonal antibody of the same clone was specified and used by many other authors as a marker of macrophages with activated phagocytosis (Dijkstra et al., 1985; Hu et al., 2007; Lee and Zhang, 2012). Similarly, MHC-II is strongly expressed in activated and phagocytic macrophages (Lee and Zhang, 2012). Our results are in line with earlier reports showing a high level of MHC-II in epiplexus macrophages (Li and Barres, 2018). There is growing body of recent evidence that inflammation in CP leads to CSF hypersecretion following the intraventricular hemorrhage (Karimy et al., 2017). Moreover, epiplexus cell activation in rat CP characterized by increased number of CD68+ and Iba-1+ cells as well as Iba-1+ cell soma size was associated with the development of hydrocephalus 24 h after SAH induction (Wan et al., 2019). Similarly, inflammation in the CP may lead to hypersecretion of CSF and thus contribute to the development of post-hemorrhagic hydrocephalus.

It has been reported that the recruitment of monocytes after SAH is stimulated by the pro-inflammatory and chemotactic molecules of the CP via activation of toll like receptor 4 (TLR-4; Meeker et al., 2012; Gram et al., 2014). Moreover, upregulation of TLR-4 by heme leads to increased TNF-α expression, inducing alterations in tight junction proteins like zonula occludens-1 (Zeni et al., 2007; Simard et al., 2009, 2011; Aveleira et al., 2010; Lin et al., 2012). Since the percentage of proliferating ED1+ cells was approximately the same in the CP of the SAH, ACSF and naïve groups, it can be assumed that their increased number in the epiplexus position probably arises from recruited monocytes. Moreover, we found a significantly increased percentage of proliferating (Ki-67+) ED2+ macrophages in the epiplexus position 7 days after SAH, but the number of ED2+ macrophages was approximately the same at all time-points. Therefore, activation and proliferation of resident ED2+ macrophages may be another source of increased number of ED1+ activated macrophages 7 days after SAH as has been previously described after nerve injury, focal brain ischemia and traumatic brain injury (Hu and McLachlan, 2003; Hu et al., 2012; Wang et al., 2013). It was demonstrated that circulating monocytes migrate through the fenestrated capillaries into the connective tissue of the CP and pass through epithelial cells by “emperipolesis” to become KC (Ling et al., 1998). Blood circulation in meningeal vessels could be another source of the increased number of epiplexus macrophages (Wilson et al., 2010).

In general, the distinct functional macrophage phenotypes are designated as M1 and M2 (Demeestere et al., 2015). M1 macrophages are characterized by expression of CD68 (ED1) and CCR7 besides other antigens (Badylak et al., 2008; Ma et al., 2010). In contrast, the expression of CD163 (ED2) and CD206 antigens are indicative of the M2 macrophage phenotype (Kigerl et al., 2009). The pro-inflammatory M1 macrophages are associated with the initial phases of acute inflammation, while anti-inflammatory M2 macrophages are associated with tissue repair and regeneration (Chazaud, 2014; Demeestere et al., 2015). It is known that modulation of immune-cell phenotype and function in the CP acts as a “checkpoint” for peripheral immune cells invading into the central nervous system (CNS) in a variety of neurological diseases (Brendecke and Prinz, 2015; Goldmann et al., 2016). Moreover, the CP has been described as the entry point for anti-inflammatory M2 macrophages into the injured spinal cord while the pro-inflammatory M1 macrophages were determined to arise from the adjacent spinal cord leptomeninges (Shechter et al., 2013). However, almost all macrophages in the CP following a cortical ischemic stroke, expressed M1-like markers indicating their pro-inflammatory immunophenotyping (Ge et al., 2017).

In our experiments, we found an increased number of CCR7+ cells (approximately one fourth of all ED1+ cells) in the CP mainly 1 day after SAH, while the cell number dropped back to that of naïve animals 7 days. In contrast, CD206+ cells gradually increased over the period of survival with a peak between 3 and 7 days. This difference in the numbers of CCR7+ and CD206+ cells is probably associated with the acute inflammatory reaction in the CP immediately after and the initiation of regenerative processes in later periods after SAH. This gradual phenotypic shift toward anti-inflammatory and cytoprotective CD206+ cells in later stages after SAH could be mediated by the induction of heme oxygenase-1 (HO-1) stimulated by hemoglobin-degradation products (Abraham and Drummond, 2006; Naito et al., 2014).

### Increased Intracranial Pressure Contributes to Immune Cell Changes in the Choroid Plexus

Increased ICP following the application of ACSF mimics aneurysmal subarachnoid hemorrhage (Prunell et al., 2002). ACSF application is widely used as a control for intrathecal drug application and experimental models of inflammatory diseases without any evidence of immunogenicity (Hylden and Wilcox, 1980; Hernangómez et al., 2016). Cellular changes found in the CP after ACSF application are therefore most likely induced by increased ICP. Our quantitative analysis revealed similarly increased numbers of ED1+ and MHC II+ cells following ACSF application as those seen in SAH animals. On the other hand, the initial increase in CCR7+ and CD206+ cell number followed by a decrease at later time points is probably a reaction to the surgical procedure. These results indicate that the reaction of epiplexus cells after SAH can also be affected by increased intracranial pressure. However, blood application (SAH group) induced a stronger immune reaction as expressed by increased number of ED2+, CD206+ cells and increased proliferation of ED2+ cells. This clearly indicates that blood and/or blood degradation products contributes to strong immune reaction in the later phase (3 and 7 days) following SAH compared to ACSF application. Since anti-inflammatory M2 macrophages (ED2+, CD206+) are associated with tissue repair and regeneration (Chazaud, 2014; Demeestere et al., 2015) we assume that this reaction may contribute to phagocytosis of the blood clot and restoration of brain homeostasis. Moreover, in contrast to the CP of naïve rats, ACSF application into the subarachnoid space elicits significantly increased epiplexus cell proliferation in the initial stages (1 and 3 days after ACSF application), but returns to normal 7 days after injection. We can assume that increased ICP may also affect the number of epiplexus immune cells in the CP by modulating cell proliferation early as an acute reaction. However, based on our results we assume that increased intracranial pressure itself does not have the ability to induce long-lasting changes in the CP.

Interestingly, we did not find CD3+ cells in any animal group even after sensitive CD3 immunofluorescence staining using cryostat sections and image analysis. In addition, positive immunostaining of spleen sections confirmed the function of the anti-CD3 antibody used. Absence of CD3+ cells in our CP samples is consistent with the finding that only a few CD3+ cells are located to the CP of naïve mice (Petito and Adkins, 2005) and indicate no recruitment of CD3+ cells following SAH or ACSF application. In addition, absence of CD3+ cells in CP following SAH might be explained by inhibitory mechanisms of T cell activation and proliferation that get triggered. It is well known that degradation of heme leads to the induction of HO-1 expression in macrophages and dendritic cells. Moreover, overexpression of HO-1 downregulates co-stimulatory molecules of dendritic cells that are necessary for the activation of T lymphocytes (Chauveau et al., 2005; Hull et al., 2014).

## Conclusion

Our experimental results demonstrate increased numbers of immune cells with diverse immunophenotypes predominantly in the epiplexus position of the CP in response to SAH. Moreover, this increased epiplexus cell number was also found in the CP of the ACSF group, indicating that not only blood, but also increased ICP contributes to cellular changes in the CP. Immune cell changes in the CP may alter the B-CSF barrier with the consequent development of hydrocephalus in response to SAH. On the other hand, SAH triggers the recruitment of epiplexus macrophages that may ingest the blood clot by phagocytosis and may thus contribute to the restoration of brain homeostasis.

## Data Availability Statement

The datasets generated for this study are available on request to the corresponding author.

## Ethics Statement

The animal study was reviewed and approved by the Animal Investigation Committee of the Faculty of Medicine, Masaryk University, Brno.

## Author Contributions

PS and MJ designed the research, performed the experiments, and wrote the manuscript. IK performed the experiments, prepared the samples for immunohistochemistry, and wrote the manuscript. PD and RJ analyzed the data and wrote the manuscript.

## Conflict of Interest

The authors declare that the research was conducted in the absence of any commercial or financial relationships that could be construed as a potential conflict of interest.
